# Schmahmann’s syndrome - identification of the third cornerstone of clinical ataxiology

**DOI:** 10.1186/s40673-015-0023-1

**Published:** 2015-02-27

**Authors:** Mario Manto, Peter Mariën

**Affiliations:** Unité d’Etude du Mouvement, FNRS-ULB Erasme, 808 Route de Lennik, 1070 Bruxelles, Belgium; Clinical and Experimental Neurolinguistics, Vrije Universiteit Brussel, Pleinlaan 2, 1050 Brussels, Belgium; Department of Neurology and Memory Clinic, ZNA General Hospital Middelheim, Lindendreef 1, 2020 Antwerp, Belgium

**Keywords:** Cerebellar cognitive affective syndrome, Posterior fossa syndrome, Cognition, Executive functions, Spatial cognition, Language, Affect, Emotion

## Abstract

Schmahmann’s syndrome represents a novel clinical condition consisting of a constellation of cognitive and affective deficits following cerebellar disease. The complex was first described in 1998 as cerebellar cognitive affective syndrome (CCAS) on the basis of a careful neurological examination, detailed bedside mental state tests, neuropsychological investigations and anatomical neuroimaging of a group of 20 patients with focal cerebellar disorders. The syndrome was characterized by four clusters of symptoms including: (a) impairment of executive functions such as planning, set-shifting, verbal fluency, abstract reasoning and working memory, (b) impaired visuo-spatial cognition, (c) personality changes with blunting of affect or abnormal behaviour, and (d) language deficits including agrammatism, wordfinding disturbances, disruption of language dynamics and dysprosodia. This complex of neurocognitive and behavioural-affective symptoms was ascribed to a functional disruption of the reciprocal pathways that connect the cerebellum with the limbic circuitry and the prefrontal, temporal and parietal association cortices. With the introduction of Schmahmann’s syndrome, clinical ataxiology has found its third cornerstone, the two others being the cerebellar motor syndrome (CMS) mainly delineated by the pioneer French and English neurologists of the 19^th^ and early 20^th^ century, and the vestibulo-cerebellar syndrome (VCS) consisting of ocular instability, deficits of oculomotor movements and ocular misalignment.

## Background

A wealth of current evidence derived from detailed neuroanatomical investigations, functional neuroimaging studies and in-depth neuropsychological assessments of patients with cerebellar disorders has brought to the fore that the cerebellum is not only implicated in sensorimotor function but also plays a cardinal role in the modulation of cognitive and affective processes [1]. The cerebellum has been considered as a pure motor controller for many decades and neurological examination of cerebellar disorders has long been exclusively directed to oculomotor disturbances, speech motor deficits, limbs incoordination and postural/gait difficulties [2]. The cerebellar motor syndrome (CMS; focusing on limb deficits) and the vestibulo-cerebellar syndrome (VCS; focusing on oculomotor deficits) have now found a third cornerstone: Schmahmann’s syndrome consisting of cognitive and affective deficits due to cerebellar disease. The aim of this editorial is to put this new syndrome into the context of cerebellar evaluation for clinicians. We briefly review the anatomy of the cerebellum, summarize the CMS and VCS, and subsequently report on the third milestone reached by J.D. Schmahmann.

### Brief anatomy of the cerebellum

**Figure 1:**
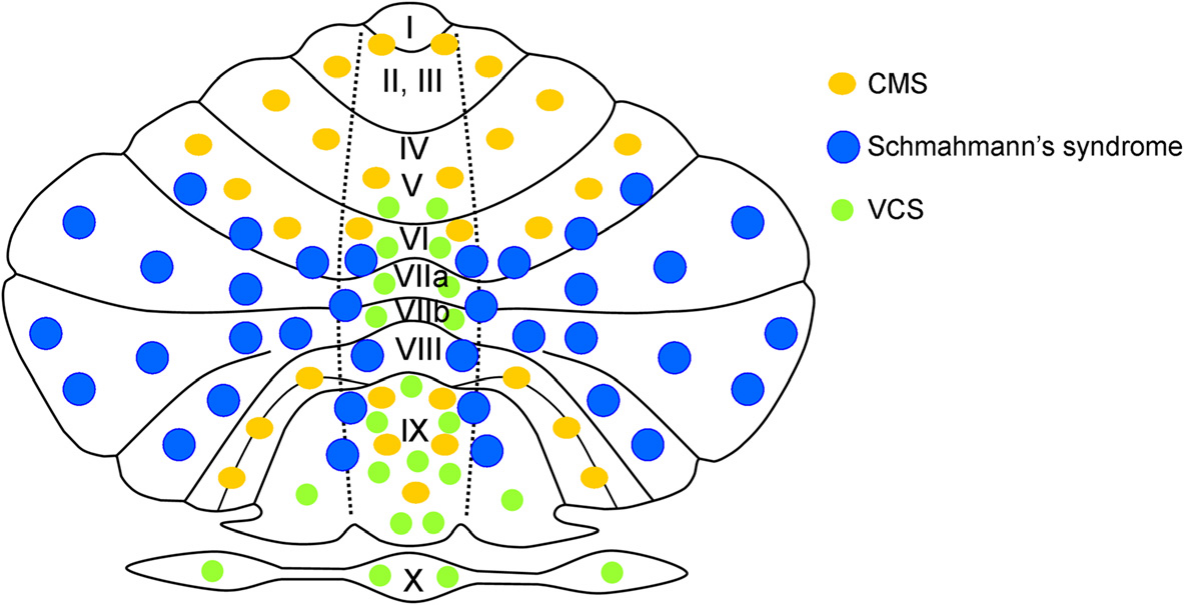
**Illustration of an unfolded cerebellum with 10 lobules (I to X according to Larsell’s classification).** Areas involved in the cerebellar motor syndrome (CMS), in Schmahmann’s syndrome and in the vestibulo-cerebellar syndrome (VCS) are indicated with orange ellipses, blue circles and green circles, respectively. The 3 fundamental syndromes underlying clinical ataxiology show a distinct profile in terms of symptom-lesion mapping. The 3 areas cover the entire cerebellum.

The cerebellum is divided in three lobes: the anterior lobe, the posterior lobe and the flocculo-nodular lobe. The anterior fissure demarcates the first two lobes. The posterolateral fissure is located between the posterior lobe and the flocculo-nodular lobe. Larsell subdivided the cerebellum into 10 lobules (I to X; see Figure 1): the anterior lobe is composed of lobules I-V, the posterior lobe includes lobules VI-IX and the flocculo-nodular lobe corresponds to lobule X [3,4]. Although there is some variability in the shape of the cerebellum between subjects, the 10 lobules are always identified [5]. There is usually an asymmetry in size between the two cerebellar hemispheres, with the left hemisphere being larger than the right one in most cases. Larsell’s nomenclature has major impacts in terms of understanding symptom-lesion mapping in cerebellar disorders.

**Figure 2:**
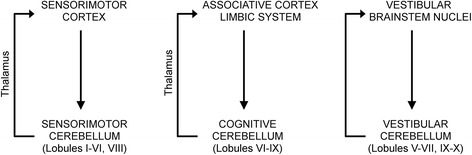
**Distinct connectivity of the sensorimotor cerebellum, the cognitive cerebellum and the vestibular cerebellum.**

Although the cerebellum is highly stereotyped in terms of neuronal circuitry, it is characterized by a functional compartmentalization due to specific input–output pathways for each cerebellar region. Connections between the cerebellum and the cerebral cortex are segregated in re-entrant loops running in parallel [6] (Figure 2):

- a primary sensorimotor region is located in the anterior lobe and the adjacent part of lobule VI. A second sensorimotor region is located in lobule VIII. Motor-related cortices project to the caudal half of the pons, which projects itself to the contralateral anterior lobe. The anterior lobe projects back to the motor cortices via thalamic nuclei.

- the posterior lobe (lobule VI, lobule VIIA which includes crus I and crus II, lobule VIIB) is considered the cognitive cerebellum. The association cortices of the cerebrum project to the medial rostral pons for the prefrontal fibers, and to the dorsal/lateral ventral pontine nuclei for the posterior fibers. Pontine nuclei project themselves to the posterior lobe. In addition, the vermis includes zones connected with the limbic system subserving emotion. The posterior lobe of the cerebellum projects back to the cerebral cortex with a relay in thalamic nuclei.

- the flocculo-nodular lobe receives afferent projections from the vestibular nuclei. Three main areas have been identified: the flocculus-paraflocculus, the nodulus-ventral uvula (lobules IX and X), and the dorsal oculomotor vermis (lobules V–VII) underlying fastigial oculomotor region. The vestibulo-cerebellar and vestibulo-spinal systems are closely linked.

Fastigial nuclei, interpositus nuclei and dentate nuclei are the main targets of the Purkinje neurons. For the sake of clarity, we will not review the distribution of mossy fibers and climbing fibers.

### The cerebellar motor syndrome (CMS)

The CMS gathers cerebellar (ataxic) dysarthria, limb ataxia and postural/gait deficits [2]. The commonality between these symptoms is a lack of motor coordination between muscles resulting in undershoot or overshoot of the intended target position [7-9]. The following clinical maneuvers and directives may be used for clinical assessment:

- cerebellar/ataxic dysarthria: phonetic quality of motor speech production is evaluated by means of formal dysarthria tests and by analysis of the phono-articulatory quality of conversational speech
- limb ataxia: ataxic disorders are identified by finger-to-finger test, finger-to-nose test, pronation/supination alternating movements, Stewart-Holmes manoeuver, knee-tibia test (heel-shin) and heel-to-knee test
- postural/gait deficits: standing capacities, body sway, gait speed and walking capacities are evaluated to identify gait and postural disorders.

A number of formal instruments are currently used to quantify motor deficits in ataxic disorders. These include the International Cooperative Ataxia Rating Scale (ICARS) [2], the Scale for the Assessment and Rating of Ataxia (SARA) [10] and the Brief Ataxia Rating Scale (BARS) [11]. ICARS is a 100-point scale and consists of 19 items. It is a validated and useful tool to quantify cerebellar motor function but due to its length of administration its use in routine clinical settings is limited. SARA is a much shorter, validated 8-item, 40-point scale. The BARS is a validated, easy to administer and reliable 30-point scale that was based on a modification of the ICARS. It consists of a 5-item subset of tests specially designed for clinical purposes. These scales include sub-scores related to the evaluation of CMS.

### The vestibulo-cerebellar syndrome (VCS)

Patients with VCS typically complain of dizziness, vertigo and imbalance [12]. The oculomotor deficits which form intrinsically part of the VCS are evaluated by means of:

- assessment of the position of the ocular globes
- testing of the ocular pursuit
- testing of saccades
- testing of vestibulo-ocular reflexes

Table 1 lists the neurological signs of the VCS as a function of the region involved [13,14].

**Table 1:** **Symptom-lesion mapping of the vestibulo-cerebellar syndrome**

**Area involved**	**Symptom**
Oculomotor vermis	Hypometric saccades
	Impaired smooth pursuit
Fastigial nucleus	Square waves
	Hypermetric saccades
	Impaired smooth pursuit
	Saccadic oscillations
Nodulus/uvula	Nystagmus (DBN^1^, PAN^2^)
	Skew deviation
Flocculus/paraflocculus	Nystagmus (DBN^1^, GEN^3^)
	Impaired smooth pursuit
	Impaired VOR^4^

Abbreviations: ^*1*^ *DBN* down-beat nystagmus, ^*2*^ *PAN* periodic alternating nystagmus, ^*3*^ *GEN* gaze-evoked nystagmus, ^*4*^ *VOR* vestibulo-ocular response.

As for CMS, ataxia rating scales include sub-scores for the quantification of VCS. Both the ICARS and BARS evaluate oculomotor movements. SARA has been criticized for the absence of oculomotor assessment.

### Schmahmann’s syndrome

As early as the 19th century a number of clinical case descriptions already suggested a possible association between congenital cerebellar pathology and cognitive as well as affective disturbances (e.g. [15]) but a possible correlation was dismissed for many decades. During the past 25 years the traditional and restricted view of the cerebellum solely as a coordinator of motor function substantially extended to that of a crucial modulator of neurocognition and affect. Following a wealth of neuroanatomical, experimental and clinical evidence in support for a cerebellar contribution to nonmotor cognition and affect, the introduction of the concept ‘cerebellar cognitive affective syndrome’ (CCAS) in the late 1990s by J.D. Schmahmann opened an entirely new area in behavioural neuroscience and established a fundamental role of the cerebellum in the modulation of neurocognition and affect [16]. In a prospective study of 20 patients with etiologically different pathologies confined to the cerebellum, Schmahmann and Sherman [16] concluded from neurobehavioural assessments that there is a typical constellation of cerebellar induced deficits which they termed CCAS (Schmahmann’s syndrome). Schmahmann’s syndrome is characterised by a cluster of multi-modal disturbances including: 1) executive deficits (deficient planning, set-shifting, abstract reasoning, working memory, and decreased verbal fluency), 2) disruption of visuo-spatial cognition (visuo-spatial disorganization and impaired visuo-spatial memory), 3) personality changes (flattening or blunting of affect, and disinhibited or inappropriate behaviour), and 4) linguistic impairments (dysprosodia, agrammatism, mild anomia). However, analysis of the clinical data revealed that not all deficits occurred in each patient but certain symptoms were particularly prominent. Decreased verbal fluency, which did not relate to dysarthria, was said to be present in 18 out of the 20 patients. Visuo-spatial disintegration, mainly consisting of disruption of the sequential approach to drawing and conceptualization of figures was found in 19 cases. Eighteen out of the 20 patients presented with executive dysfunctions involving working memory, motor and mental set-shifting and perseverations of actions and drawing. In 15 patients frontal-like behavioural and affective changes were evident. Flattening of affect or disinhibition occurred, taking the form of overfamiliarity, flamboyant and impulsive actions, and humorous but inappropriate comments. Behaviour was characterized as regressive and child-like in some cases and obsessive–compulsive traits were occasionally observed. Deficits in mental arithmetic was evident in 14 patients. Visual confrontation naming was impaired in 13 patients. Eight patients developed abnormal prosody characterised by high pitch, whining and a hypophonic speech quality. Mnestic deficits (verbal and visual learning and recall) were observed in some. The cluster of symptoms defining the Schmahmann’s syndrome was associated with a decrease of general intellectual capacity.

From an anatomo-clinical perspective, cognitive and affective impairments were more prominent and generalized in patients with large, bilateral, or pancerebellar disorders, especially in a context of an acute onset of cerebellar disease. Posterior lobe damage was particularly important in the genesis of this novel syndrome. Damage of the vermal regions was consistently present in patients with disruption of affect. Anterior lobe damage was found to be less important to cause cognitive and behavioural deficits. The Schmahmann’s syndrome in patients with stroke improved over time, but executive function remained abnormal.

Schmahmann and Sherman [16] pointed out that on the basis of their observations it was not possible to distinguish the contribution of the lesioned cerebellum to these abnormal behaviours from that of the cerebral regions newly deprived of their connections with the cerebellum. Indeed, the clinical features of the cognitive and affective impairments constituting Schmahmann’s syndrome are identical to those usually identified in patients with supratentorial lesions affecting the cortical association areas and paralimbic regions and their interconnections. For example, socially inappropriate behaviour, personality changes and affective alterations typically result from frontal lobe damage. Reciprocal neuroanatomical connections linking the cerebral association areas and paralimbic regions with the cerebellum constitute the basis to explain the pathophysiological mechanisms of the cerebellar induced cognitive and affective deficits. As pointed out by Schmahmann and co-workers in an influential series of neuroanatomical studies, the cerebrocerebellar anatomical circuitry consists of a feedforward limb (the corticopontine and pontocerebellar pathways) and a feedback limb (the cerebellothalamic and thalamocortical systems) reciprocally connecting the cerebellum with the supratentorial regions crucially implicated in cognitive and affective processing. Modern non-invasive techniques such as transcranial magnetic stimulation (TMS) and transcranial direct current stimulation (tDCS) allow to explore the causal role of the cerebellum in abnormal behaviours and enrich our understanding of the contributions of the cerebellum in the cerebrocerebellar circuits by modulating the connectivity between the cerebellum and cerebral cortices [17,18]. These techniques might be helpful to elucidate the specific roles of distinct anatomical regions of the cerebellum.

Schmahmann’s syndrome has been amply documented in a high number of publications describing both children and adults with etiologically heterogeneous congenital, neurodevelopmental and acquired neurological and psychiatric disorders (primarily) affecting the cerebellum. Within a large clinical spectrum of mild to severe cerebellar induced cognitive and affective dysfunctions the “posterior fossa syndrome” (PFS), also known as “cerebellar mutism syndrome” (e.g. [19]) might be regarded as a semiological subtype of Schmahmann’s syndrome [20]. In this syndrome a complete loss of speech dynamics (verbal mutism) is accompanied by (frontal-like) neurobehavioural abnormalities including apathy, loss of drive or reduced initiative, unconcern, inconsolable crying and whining. Transient speechlessness is generally considered to be the hallmark feature of the PFS but mutism occasionally does not materialize at all and a wide range of postoperative neurobehavioural deficits entirely consistent with Schmahmann’s syndrome (e.g. [21]) may be found when carefully looked for.

Current methods for neurobehavioural assessment include a wide variety of standardised neuropsychological test batteries and questionnaires primarily designed to investigate the integrity of cognitive and affective functions at the supratentorial level (e.g. Wechsler’s Intelligence and Memory Scales). As a result, a range of subtle cognitive and affective impairments following disruption of the modulatory role of the cerebello-cerebral network in cognition and affect might go unnoticed using these gross assessment tools. Unlike CMS and VCS, there is still no single scale or clinical instrument that is validated and easy to administer in order to reliably assess cerebellar induced cognitive and affective dysfunctions constituting Schmahmann’s syndrome. Further studies are required.

## Conclusion

Since the introduction of Schmahmann’s syndrome at the end of the 1990s, clinical ataxiology has been enriched with its third cornerstone. The development of this novel clinical syndrome substantially expanded the role of the cerebellum to that of a crucial modulator of neurocognitive and affective processing through an integrated network of cerebello-cerebral connections. CMS, Schmahmann’s syndrome and VCS are congruent with the modular organization of cerebellum, confirming that a careful clinical observation meets the neuroanatomy.
